# 

**Published:** 1891-01

**Authors:** 


					﻿ICONTENTS
PAGE. I
The New Year................... 1	|
Consumption Cured by Subcu-
taneous Injections............ 1
The Hygiene of Motherhood ...	4	|
Coffee Drinking................. 8	i
The Corset...................... 8	’
How to Treat the Ear............ 9
Statistics of Interest.......... 9
The Fly a Carrier of Micro-or-
ganisms ...................... 10
Who Pulled the Bell-rope t .... 12
Disinfection by Sulphur....... 13
The Hope of Immortality...... 14
The Sewage Farms of Berlin... 15
Dietary .....................   17
Treatment of Fever............ 18
Miscellaneous ................  19
Harvey’s Wonderful Power... . 20
Dining in Lapland............. 21
Literary..................... 22
Our Birth..................... 24
OF
/^uiLF A PAGE ANb\S^k.
Ho^Jo^j^ett^Hg^Shate/C. I
Lydia E. Pinkham Med.,Co.. II
Rochester Lamp Co......... Ill
Hall’s Journal of Health. V
Hall's Journal Premiums... VI
Ayer’s Cathartic Pills ..  VII
Stillman Remedies Co......IX,	X
Herba Vita Remedy Co...... XI
Tribune Chemical Co.......XIII
O. S. Trifet.............. XIV
Royal Baking Powder....... XV
S. Johnson & Co............ XV
				

